# Hi-C untangles the temporal dynamics of the children’s gut resistome and mobilome, highlighting the role of transposable elements

**DOI:** 10.1128/mbio.01134-25

**Published:** 2025-08-12

**Authors:** Sara G. Cifuentes, Jay Graham, Gabriel Trueba, Paúl A. Cárdenas

**Affiliations:** 1 Instituto de Microbiología, Colegio de Ciencias Biológicas y Ambientales, Universidad San Francisco de Quito USFQ 603002 , Quito, Pichincha, Ecuador; 2 Berkeley School of Public Health, University of California 40289, Berkeley, California, USA; Instituto de Biologia Molecular y Celular de Rosario, Rosario, Santa Fe, Argentina

**Keywords:** antibiotic resistance, mobile genetic elements, transposable elements, Hi-C metagenomics, gut microbiota

## Abstract

Many metagenomic studies lack the ability to measure the temporal dynamics of the intestinal resistome (the collection of antibiotic resistance genes [ARGs]) and mobilome (the collection of all mobile genetic elements that enable their transfer) and link the genetic features to specific species in the gut. We applied Hi-C sequencing and shotgun metagenomics to study fecal matter from children (*n *= 15) living in semi-rural communities of Quito, Ecuador. We sampled at three different periods, with a 4- to 6-month interval between each sample collection. To understand the dynamics of ARGs from different genetic perspectives, we focused on identifying classes of mobile ARGs that are classified as high risk to human health. We selected those ARGs that appeared at least twice across sampling periods in the same child and focused the longitudinal analysis on the subset of children (*n *= 6) where these high-risk ARGs were consistently detected. The study demonstrated the temporal dynamics of these mobile ARGs from the taxonomic, plasmid, and transposable element perspectives, including insertion sequences and transposons. Our findings reveal that while plasmid composition fluctuates over time, transposons play a crucial role in the stability and dissemination of ARGs. Specifically, *aph(3″)-Ib* and *aph(6)-Id* genes were consistently mobilized by transposons across multiple multidrug-resistant *Escherichia coli* strains. These results highlight the importance of transposons in shaping the gut resistome and suggest that tracking regionally significant transposons could improve our understanding of ARG transmission in small geographic areas.

**IMPORTANCE:**

Antibiotic resistance (ABR) is a growing global challenge, and particularly high-risk antibiotic resistance genes (ARGs) are a threat to public health. While plasmids are often considered the cornerstone of the spread of ARGs, our study emphasizes the critical role of transposons in the persistence and mobility of ARGs within the gut microbiota. By integrating Hi-C sequencing and shotgun metagenomics, we show that transposons mediate the transfer and persistence of ARGs across different *Escherichia coli* lineages, while plasmid composition changes over time. Recognizing the impact of transposons on resistome dynamics can help refine strategies to mitigate ABR transmission, particularly in regions where the impact of resistance is most significant, such as low- and middle-income countries. Our findings provide new insights into the mechanisms driving the persistence of ABR in the human gut, which are essential for developing more effective public health interventions and incorporating transposable elements into surveillance efforts.

## OBSERVATION

Antibiotic resistance (ABR) is a global health threat (1), primarily driven by the dissemination of antibiotic resistance genes (ARGs) through mobile genetic elements (MGEs), including plasmids and transposable elements (TEs) (2, 3). While plasmids are key ABR vectors, TEs (4) mobilize ARGs between plasmids. High-risk ARGs, which are highly mobile and pose a significant public health risk (5, 6), persist and spread via these MGEs. High-throughput chromosome conformation capture (Hi-C) is a recently developed technique that links ARGs, plasmids, and other MGEs to their microbial host *in situ* by preserving spatial proximity (7). Hi-C overcomes some of the limitations of shotgun sequencing and culture-based methods (7, 8), but few longitudinal studies have evaluated the temporal dynamics of mobile ARGs in human populations (9).

We integrated Hi-C and shotgun metagenomics to study the mobility and host associations of ARGs over time in the gut microbiota of 15 healthy children (4 months to 5 years) from semi-rural communities in Quito, Ecuador. Samples were collected at three time points (4–6 months apart) to analyze temporal changes in ARGs, MGEs, and bacterial hosts. Study design, sample collection, and inclusion criteria were previously documented in a longitudinal (10) and a metagenomics study (11). DNA extraction, sequencing, and bioinformatics protocols for Hi-C and shotgun metagenomics have been detailed by Cifuentes et al. (12). This approach identified which TEs or plasmids carry ARGs and in which type of bacteria they were found.

**Fig 1:**
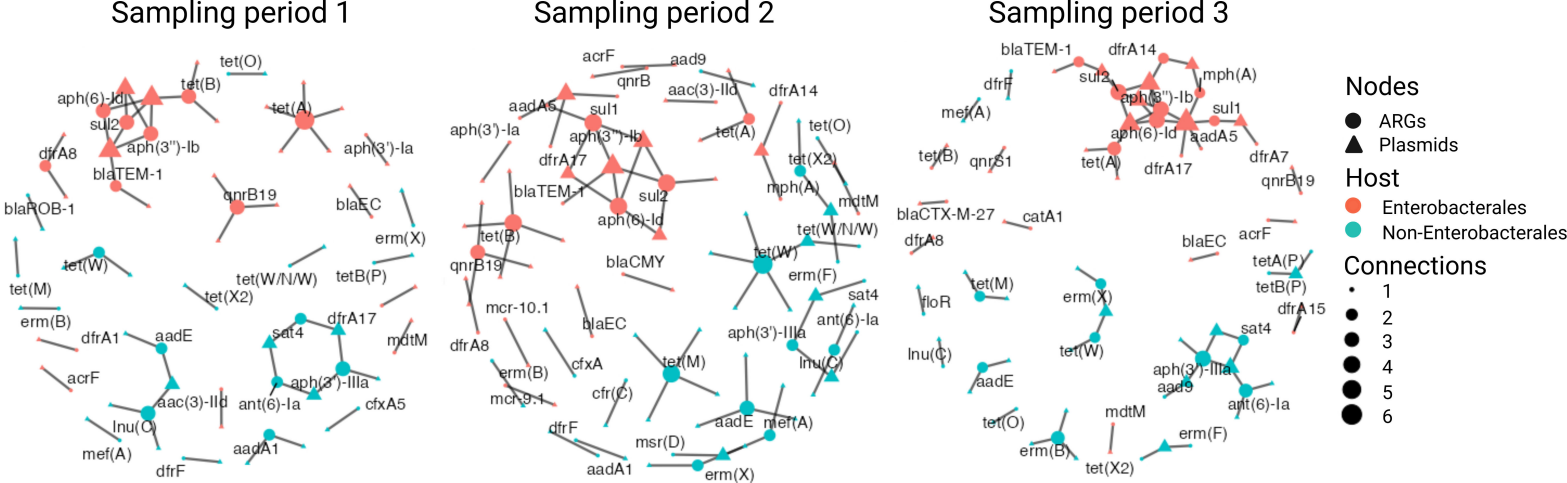
Temporal dynamics of mobile ARGs, plasmids, and bacterial hosts across three sampling periods. This network diagram (ggplot2 package, v.3.5.1) illustrates the connectivity of ARGs (circles) and plasmids (triangles) with bacterial hosts over time. Each triangle represents a unique plasmid in the network layout for visualization purposes; identical plasmids may exist in multiple hosts or samples but they are represented as separate nodes to simplify network interpretation. Node colors represent bacterial classification: orange for Enterobacterales and fuchsia for non-Enterobacterales. Node size corresponds to connectivity, with larger nodes indicating ARGs with higher mobility potential. The network is presented at the community level to reveal broad temporal trends in ARG mobility and host associations rather than child-specific dynamics. Fecal samples were collected from 15 children aged 4 months to 5 years, with sampling intervals of 4–6 months. Due to age variability across participants, individual-level differences are explored in more detail in Fig. 2.

The network diagram in Fig. 1 illustrates the temporal dynamics of mobile ARGs and their association with plasmids and bacterial hosts, including Enterobacterales (e.g., *Escherichia coli*, *Enterobacter asburiae*) and non-Enterobacterales (e.g., Clostridiales, *Bifidobacterium breve*). The lack of plasmid transfer between bacterial phyla in our study is consistent with previous findings reporting that approximately 80% of MGEs transfer between genera but rarely between phyla (13). Although Fig. 1 does not differentiate between commensal and pathogenic *E. coli* lineages*,* prior research indicates that MGE-mediated ARG exchange occurs between both (13).

Furthermore, the dense ARG networks observed among Enterobacterales, particularly *E. coli*, emerged from the data analysis rather than being predefined. These bacteria consistently harbored the most ARGs across all time points. Previous studies analyzing the infant gut resistome (14, 15) reported that high abundance of ARGs was correlated with the presence of *E. coli*. These conclusions were based on shotgun metagenomics and taxonomic profiling, which inferred associations based on relative abundances but could not directly link ARGs to their bacterial host. The use of Hi-C metagenomics evidences the physical relationship among ARGs with specific taxa, corroborating the significant contribution of *E. coli* to the gut resistome in infants and preschoolers (4 months to 5 years). Furthermore, Fig. 1 shows that the ARG-plasmid co-occurrence patterns varied over time, evidencing the dynamic nature of the gut resistome.

We identified several ARGs with a higher degree of mobility, as indicated by their presence on three or more different plasmids (in our network analysis). These include *tet(A*), *tet(B*), *tet(M*), *tet(W)* (tetracycline resistance), *lnuC* (lincosamide resistance), *qnrB19* (quinolone resistance), *sul2* (sulfonamide resistance), *aadE*, *ant(6)-Ia*, *aph(3′)-IIIa*, *aph(3″)-Ib*, *aph(6)-Id* (aminoglycoside resistance), and *ermB* (macrolide resistance). These ARGs exhibit broad host ranges and frequent co-occurrence with multiple plasmid types, characteristics associated with high dissemination potential in clinically relevant pathogens. Additionally, ARGs such as *sul2, aph(3″)-Ib,* and *aph(6)-Id* appeared repeatedly across sampling periods, possibly due to their longer circulation in human-associated bacterial communities (16). In contrast, ARGs conferring resistance to recently used antibiotics, such as *bla*_CTX-M-27_, *mcr-9.1*, and *mcr-10.1,* were not consistently observed across time points in the gut of the same children (i.e., sampled every 4–6 months). These ARGs have recently emerged, and previous studies have attributed their spread to selective pressure resulting from the intensive use of latest generation antibiotics in human healthcare and food animal production (17, 18). These conclusions are based on genomic epidemiology, which links plasmid-carrying isolates across compartments. Although previous studies have shown that resistant transconjugant lineages can persist for several months without antibiotic exposure (19), our findings differ. In our cohort, we did not consistently observe persistence of recently emerged ARGs such as *mcr-9.1*, *mcr-10.1*, and *bla*_CTX-M-27_. This discrepancy may reflect differences in microbiota composition and gut ecosystem dynamics in our semi-rural population. In addition, variation in plasmid content and the dominant ARG-carrying taxa (e.g., *E. coli*) across sampling periods, as shown in Fig. 1, could influence the stability and retention of these ARGs over time (14).

**Fig 2:**
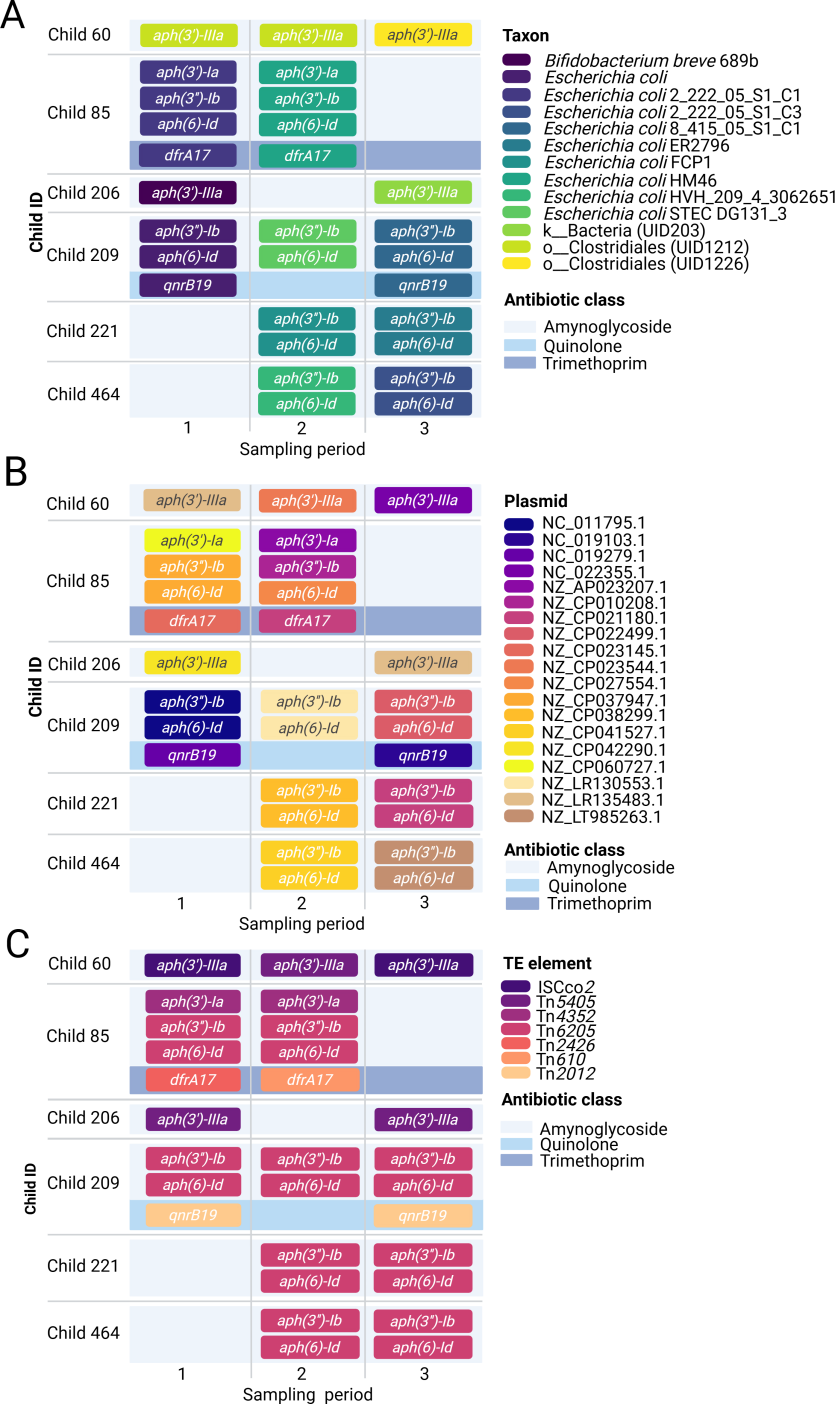
Longitudinal analysis of high-risk ARGs at the taxonomic and mobile genetic element (MGE) levels. Fecal samples from six children, collected across three sampling periods (4–6 months apart), were analyzed to track the persistence and mobility of high-risk ARGs. The temporal dynamics of ARGs are presented from three perspectives: (**A**) taxonomic associations (ARG–host relationships), (**B**) plasmid context (ARG plasmid links), and (**C**) TE context (ARG-TE associations). Children were selected based on the repeated detection of high-risk ARGs in at least two sampling periods, with complete Hi-C and shotgun metagenomic data available enabling longitudinal tracking. Children with incomplete data or without repeated ARG detection were not included. Children were aged between 1.0 to 3.6 years at the time of sampling (60: 2.1–3.0 years; 85: 1.0–1.9 years; 209: 1.2–2.1 years; 21: 1.3–2.2 years, 464: 2.7–3.6 years), allowing visualization of temporal ARG dynamics across early childhood. Data visualization was performed using BioRender (https://BioRender.com).

The host species and plasmid carrying high-risk ARGs changed at each sampling period. Fig. 2 reveals the time trajectories of high-risk ARGs appearing at least twice across all three sampling periods. We focused on *aph(3″)-Ib* and *aph(6)-Id*, co-occurring in multiple children. Fig. 2A and B show these genes coexisting in the same sampling period but within different *E. coli* lineages or plasmids at successive time points. This indicates that the ARGs were not confined to a single bacterial strain or a plasmid. Strikingly, when analyzing these ARGs from the perspective of TEs (Fig. 2C), *aph(3″)-Ib* and *aph(6)-Id* were consistently found within the same transposon (initially identified as Tn*6205* using MobileElementFinder (v.1.1.2) with a 60% coverage threshold) (20), at different sampling periods. Our findings demonstrated that in the case of the *aph(3″)-Ib* and *aph(6)-Id* genes*,* TEs seem to play a key role in the permanence of these genes, beyond plasmids or bacteria. Transposons and IS also mediate ARG transfer between plasmids and chromosomes (20, 21). Focusing on TEs redefines antibiotic resistance transmission, emphasizing their role as stable reservoirs. Unlike plasmids, transposons can integrate into diverse genomic contexts and persist across phylogenetically distant bacteria. This ability may mask direct ARG transmission routes in One Health studies, where identical ARGs appear in different species or compartments without clear evidence of shared plasmids or strains (22, 23). Therefore, transposons may act as hidden drivers of ARG dissemination, complicating efforts to trace resistance pathways across the human–animal–environment interface.

We characterized the genomic environment of the *aph(3″)-Ib* and *aph(6)-Id* genes using the previously described protocol for DNA annotation and genomic structure comparison (4). We found a *sul2* (sulfonamide resistance) gene immediately upstream of *aph(3″)-Ib–aph(6)-Id* in most assemblies, and in more than half of the cases, an MGE-associated *tnpA* transposase gene immediately downstream (Fig. S1). The *tnpA* sequence showed high identity to an IS*91*-family transposase, suggesting that the composite element belongs to the IS*91* (Tn*3*) family of replicative transposons (24). This finding is intriguing because our initial annotation (using MobileElementFinder with a 60% coverage threshold) had labeled the element as Tn*6205*, which is associated with the IS*6* family. We were unable to identify conserved right-end sequences (IRRs), likely due to the presence of truncated contigs, a known limitation of metagenomic assemblies (25). However, the *tnpA* gene is likely functional, as it aligns perfectly with known IS*91* sequences using BLASTp. The IS*91* transposase finding suggests that we may be observing a previously undescribed transposon variant that carries *sul2*, *aph(3″)-Ib*, and *aph(6)-Id* together.

This study underscores the value of advanced genomic tools in understanding gut resistome dynamics. By integrating Hi-C metagenomics with longitudinal sampling, we provide evidence of ARG transfer events and stable associations with transposons, while plasmids and bacterial hosts change. Our findings emphasize transposons as key drivers of AMR persistence, informing surveillance strategies to mitigate the spread of ABR.

## Data Availability

This Hi-C and shotgun metagenomics project has been deposited at DDBJ/ENA/GenBank as BioProject PRJNA1082298.

## SUPPLEMENTAL MATERIAL

Fig. S1 mbio.01134-25-s0001.docx Genetic environment of *aph (3')-Ib, aph(6)-Id* genes across sampling periods.

ASM does not own the copyrights to Supplemental Material that may be linked to, or accessed through, an article. The authors have granted ASM a non-exclusive, world-wide license to publish the Supplemental Material files. Please contact the corresponding author directly for reuse.
