# Supplementary material for: Hi-C untangles the temporal dynamics of the children’s gut resistome and mobilome, highlighting the role of transposable elements
Source: mBio. 2025 Aug 12;16(9):e01134-25. doi: 10.1128/mbio.01134-25 (PMC12421864; doi:10.1128/mbio.01134-25)
Supplement: Fig. S1 — Genetic environment of aph (3')-Ib, aph(6)-Id genes across sampling periods. [file mbio.01134-25-s0001.docx]

Sara G. Cifuentes^1^, Jay Graham^2^, Gabriel Trueba^1^, Paúl A. Cárdenas^1*^

1 Universidad San Francisco de Quito USFQ, Colegio de Ciencias Biológicas y Ambientales, Instituto de Microbiología, Diego de Robles St, Cumbayá, Quito, Pichincha, Ecuador

2 University of California, Berkeley School of Public Health, 2121 Berkeley Way West, Berkeley, California, USA

***Corresponding author:** Paúl A. Cárdenas

Universidad San Francisco de Quito USFQ, Colegio de Ciencias Biológicas y Ambientales, Instituto de Microbiología, Diego de Robles St, Cumbayá, Quito, Pichincha, Ecuador

E-mail: [pacardenas@usfq.edu.ec](mailto:pacardenas@usfq.edu.ec)

Phone: (+593) 2 297-1700 ext. 1517

**Hi-C Untangles the Temporal Dynamics of the Children’s Gut Resistome and Mobilome, Highlighting the Role of Transposable Elements**

**SUPPLEMENTAL MATERIAL**


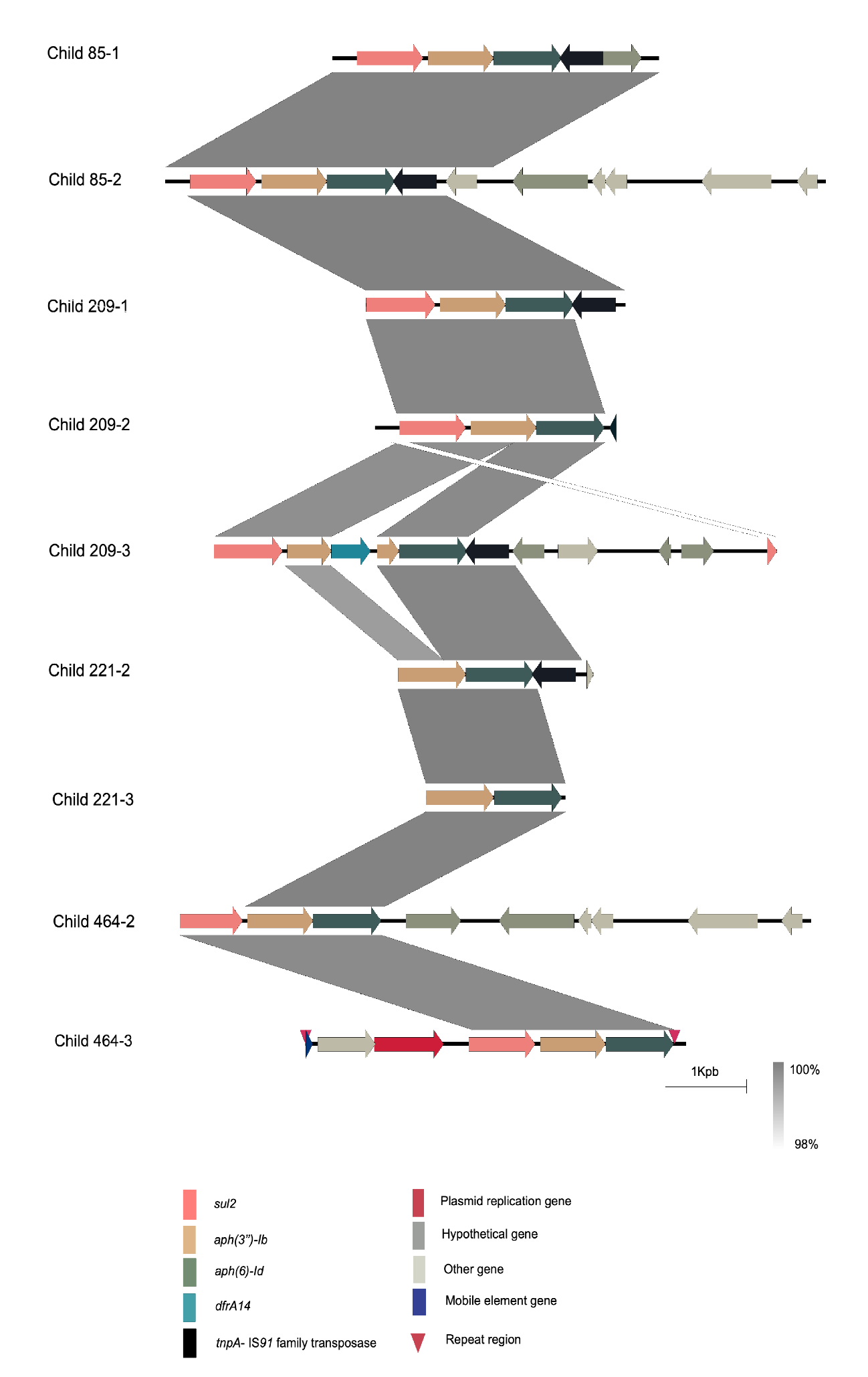


**Fig S1** Genetic environment of *aph (3’’)-Ib, aph(6)-Id* genes across sampling periods. This schematic illustrates the genomic environment of *aph(3’’)-Ib* and *aph(6)-Id* across different sampling periods. Each row represents a distinct child sample, where the number after the child ID denotes the sampling period. Gene orientations have been adjusted for samples 209-1, 221-2, and 221-3 for alignment consistency. Functional annotations include resistance genes, transposase elements, and other genomic features.
